# Characteristics of Nurse-Reported Adverse Events: An Exploration Based on FAERS Data

**DOI:** 10.1093/geroni/igaf122.2549

**Published:** 2025-12-31

**Authors:** Jieyu Mao, Miyae Yamakawa, Yasushi Takeya

**Affiliations:** Osaka University, Osaka, Osaka, Japan; Osaka University, Osaka, Osaka, Japan; Osaka University, Osaka, Osaka, Japan

## Abstract

Adverse events (AEs) are undesirable experiences linked to medical product use, particularly in aging populations with polypharmacy. Nurses play a critical role in AE reporting due to their close involvement in patient care and medication administration. This study used the FDA Adverse Event Reporting System (FAERS) database to analyze the characteristics of nurse-reported AEs and highlight nurses’ role in pharmacovigilance. Data from the Side Effect Resource (SIDER) database were integrated to assess the proportion of unknown adverse drug reactions in reported AEs, while report quality and completeness were evaluated using the VigiGrade scoring system, a method for assessing the quality of individual case safety reports. After extraction and deduplication, 8,653 nurse-submitted reports were analyzed, with the majority concentrated between 2012 and 2014, accounting for only 5% of all reports during this period. Nurses primarily reported AEs related to general disorders and administration site conditions, with a strong focus on tumor-related indications and specific therapies. The average time from event occurrence to reporting was 247.8 days, the shortest among all occupations. The proportion of reported AE symptoms not found in the SIDER database was 11.82%. Nurses achieved the highest VigiGrade scores across all dimensions, demonstrating the ability to provide high-quality AE reports. However, the role of nurses remains undervalued, restricting their ability to identify unknown ADRs and apply unique clinical perspectives. To strengthen the role of nurses, targeted training programs and policy improvements are necessary to reduce barriers and enhance their participation in AE reporting.

